# 885. Promoting Diversity, Equity and Inclusion with a quarterly virtual Grand Rounds for the Pediatric Infectious Diseases Society

**DOI:** 10.1093/ofid/ofad500.930

**Published:** 2023-11-27

**Authors:** Tanya Rogo

**Affiliations:** Brown University Alpert Medical School, Providence, Rhode Island

## Abstract

**Background:**

The Inclusion, Diversity, Access and Equity (IDA&E) Taskforce was created by the Pediatric Infectious Diseases Society (PIDS) in 2020. A 2021 survey of the PIDS membership to assess perceptions of diversity, equity and inclusion (DEI) revealed several areas of concern: perceptions of bias against and lack of support for international medical graduates (IMGs); lack of diversity in PIDS leadership; and perceived exclusivity in PIDS social and networking events. There is evidence that implicit bias can be alleviated by education that both increases awareness and provides bias reduction strategies. The Taskforce introduced a quarterly virtual Grand Rounds for the PIDS membership to provide DEI education. This abstract describes the first year of this initiative.

**Methods:**

A proposal for a quarterly IDA&E Grand Rounds was approved by the PIDS Executive Committee in February 2022. A member of the IDA&E Taskforce assumed the role of course director. Four webinars were held in March, June, and September 2022, and January 2023. The topics addressed were under-representation in Pediatric Infectious Diseases, career challenges faced by IMGs, leadership development, and career pathways within Pediatric Infectious Diseases. A feedback survey link was provided to attendees of each Grand Rounds.

**Results:**

The four Grand Rounds had an average of 172 registrants (range 130-204). Feedback survey completion was low (average 8 per webinar, range 4-18). Survey respondents reported affirmation of personal experiences; increased awareness of the topic discussed; and desire for more content related to career pathways, and IMG and visa-related concerns. Respondents commented on low attendance by division heads and fellowship program directors and recommended promotion of Grand Rounds attendance among persons in leadership positions.

**Conclusion:**

Specialty-specific DEI education can be provided at the national level by a medical society through periodic webinars. The hour-long virtual format is limited in ability to address topics in-depth and should be the bare minimum of the work needed by institutions, training programs, and individual practitioners to support workforce diversity in Pediatric Infectious Diseases.

**Disclosures:**

**Tanya Rogo, MD**, AstraZeneca: Grant/Research Support|Pfizer: Advisor/Consultant|Pfizer: Grant/Research Support

